# Walnut Protein Isolate-κ-Carrageenan Composite Gels Improved with Synergetic Ultrasound-Transglutaminase: Gelation Properties and Structure

**DOI:** 10.3390/gels9020091

**Published:** 2023-01-20

**Authors:** Yanlong Liu, Yuqing Lei, Xu Kang, Hui Ouyang, Xiuting Li, Xiongwei Yu, Qianhui Gu, Shugang Li

**Affiliations:** 1 Key Laboratory of Fermentation Engineering, Ministry of Education, School of Food and Biological Engineering, Hubei University of Technology, Wuhan 430068, China; 2 Engineering Research Center of Bio-Process, Ministry of Education, Key Laboratory for Agricultural Products Processing of Anhui Province, School of Food and Biological Engineering, Hefei University of Technology, Hefei 230601, China; 3 Food Nutrition and Safety Professional Think Tank Base of Beijing Association for Science and Technology, Beijing Technology and Business University (BTBU), Beijing 102488, China; 4 Wuhan Xudong Food Co., Ltd., Wuhan 430000, China; 5 Three Squirrels Inc., Ltd. Wuhu 241000, China

**Keywords:** ultrasound, transglutaminase, walnut protein isolate, κ-carrageenan, gel properties, structure

## Abstract

Walnut protein is a kind of natural, high-quality plant protein resource. However, its high content of gluten, strong hydrophobicity and poor gelation ability have greatly limited its development and utilization in gel products. It was found in this experiment that ultrasonic power combined with transglutaminase (TGase) had a significant effect on the gel properties of the walnut protein isolate (WNPI)-κ-carrageenan (KC) complex. The results showed that the gel strength of the WNPI-KC complex first increased and then decreased with the increase in ultrasonic power (0–400 W). WNPI-KC composite gel had the best texture properties, rheological properties, water-holding capacity (99.41 ± 0.76%), swelling ratio (2.31 ± 0.29%) and thermal stability (83.22 °C) following 200 W ultrasonic pretreatment. At this time, the gel network was more uniform and much denser, and the water molecules were more tightly bound. Further, 200 W ultrasonic pretreatment could promote the transformation of α-helices to β-folds in protein molecules, improve the fluorescence intensity, increase the content of free sulfhydryl groups and enhance the intermolecular forces. The experimental results could provide technical support for the development of walnut protein gel food.

## 1. Introduction

Walnut protein isolate (WNPI) is a by-product in the production process of walnut oil [1]. It has received widespread attention because of its high nutritional value, strong functionality and rich content [2]. Studies have shown that walnut protein food can prevent cardiovascular disease, heart disease and certain cancers [3]. Walnut protein is composed of four components, albumin, globulin, prolamin and glutenin, and their contents are 6.81%, 17.57%, 5.33% and 70.11%, respectively [4]. High gluten content and low water solubility limit the gelation properties of walnut protein. Structural properties, modification methods, processing parameters and interactions with other components have displayed great influences on the gel properties of walnut protein [5]. Therefore, understanding the influence of modification methods on the structural properties of walnut protein and the interaction between walnut protein and other components is of great significance for the development of walnut protein isolate gel products.

In the walnut protein gel system, the polysaccharide can change its gel behavior by interacting with the protein, thereby affecting the structure and gel properties of the composite gel [6]. κ-Carrageenan (KC) is a hydrophilic colloid extracted from red algae seaweed. It can complex with proteins to improve the gel strength, textural properties and microstructure of proteins, thereby changing the quality of the product. Jiang et al. found that KC significantly improved the rheological properties, textural properties and WHC of oyster protein, thereby improving its gel network structure [7]. Walayat et al. revealed that the addition of KC enhanced the function and gelling ability of grass carp myofibrillar proteins gel during 60 days of refrigerated storage [8]. These results indicated that kappa-carrageenan could interact with proteins and further affect gel properties. So, it was feasible to add KC to improve the gel properties of WNPI in this research study.

Transglutaminase (TGase) can be used to catalyze protein cross-linking, thereby improving protein gelation [9]. TGase can catalyze the acyl transfer reaction of glutamine residues and lysine residues of proteins and is popular in the production of meat products, dairy products, baked goods and other products. TGase can significantly increase the strength of dough, thereby improving the stability and quality of bread [10]. Moreover, TGase treatment could also improve the foaming, emulsifying, gel properties, thermal stability and water-holding capacity (WHC) of proteins [11]. The study found that exposing the targeted site of the enzyme could promote the cross-linking reaction of TGase and further improve the gel properties of the protein [12]. Heat treatment, ultrafine grinding treatment and ultrasonic treatment are several common methods to improve the cross-linking degree of TGase [13].

In recent years, ultrasound has received extensive attention due to its green, non-toxic and low-energy-consumption advantages. Ultrasound can interact with proteins through cavitation and mechanical effects, thereby improving the functional properties of matter [14]. Generally, ultrasonic waves are considered pressure waves at frequencies greater than 20 kHz. During sonication, the fluid forms small bubbles that burst violently, creating extremely high, localized temperatures and pressures [15]. In addition, sonication can disrupt the intermolecular or intramolecular interactions of proteins and possibly promote TGase cross-linking reactions [16]. Zhang et al. reported that ultrasound could enhance the TGase-mediated cross-linking of whey protein soluble aggregates, thereby improving their rheology and gelling properties [17]. Zhang et al. documented that ultrasound changed the physicochemical and structural properties of soybean protein isolate, resulting in a better TGase cross-linking substrate, thereby forming a better gel network structure [18].

In this study, the effects of ultrasonic pretreatment on the texture properties, rheological properties, WHC and thermal stability of TGase-cross-linked WNPI-KC composite gels were investigated. In addition, the structural properties and intermolecular forces of composite gel were also investigated. The experimental work carried out could provide a new hypothesis for improving the gel properties of WNPI, thereby promoting the high-value utilization of walnut protein.

## 2. Results and Discussion

### 2.1. Effects of Ultrasonic Power on WHC, Swelling Ratio, Freeze–Thaw Stability and Moisture Distribution of WNPI-KC Composite Gels

**Figure 1:**
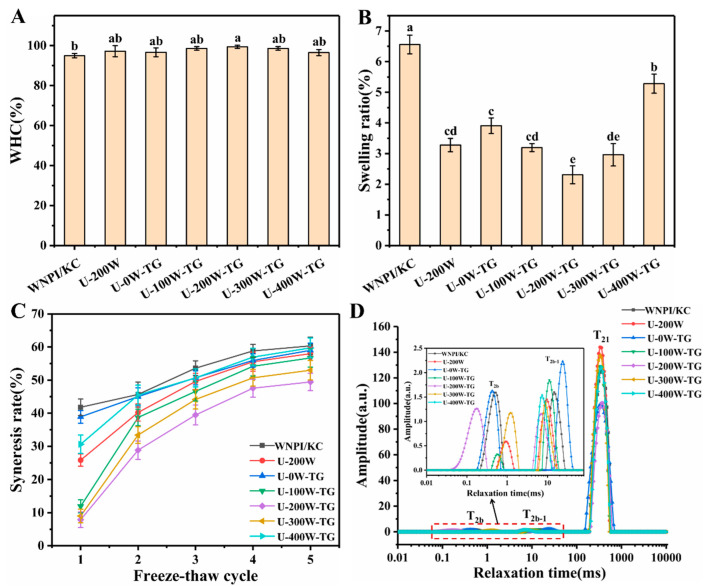
Effects of ultrasonic power on WHC (**A**), swelling ratios (**B**), freeze–thaw cycles (**C**) and moisture distribution (**D**) of WNPI-KC composite gels. Different letters on the bars indicate significant differences (*p* < 0.05).

The WHC reflects the binding capacity of gel to water and can be used to measure the quality of gel. It is well known that the WHC of composite gels is closely related to the microstructure. The more uniform and the denser the microstructure of the gel is, the better its gel strength and WHC are [19]. It can be seen in Figure 1A that after ultrasonic pretreatment, the WHCs of WNPI-KC composite gels had no significant differences, but they were higher than that of the control group. This might have been due to the fact that sonication reduced the particle sizes of WNPI and KC, which promoted TGase cross-linking and produced a tight and uniform gel network, leading to the enhancement of gel strength and WHC. Compared with other sonication groups, the WHC of the composite gel was the highest when sonicated at 200 W.

The swelling ratio is an important indicator to measure the water absorption of gel, and it can also reflect the performance of gel. The denser the network structure of the gel is, the weaker its WHC is, and the lower the swelling rate becomes. As shown in Figure 1B, after combined treatment with ultrasound-TGase, the swelling rates of the composite gel networks were significantly lower than that of the control group, which might have been due to the enhancement of covalent bonds between the gel networks induced by ultrasound-TGase, limiting the relaxation of the molecular chains, thereby preventing water from entering the network. In addition, when the ultrasonic power was 200 W, the swelling rate of the WNPI-KC composite gel was the lowest, which might have been related to its higher cross-linking density.

Freeze–thaw stability refers to the amount of dehydration of food during freezing and thawing, which can reflect the dehydration trend of food and is an important indicator of frozen food. A low dehydration value means that the gel has a higher WHC. It can be seen in Figure 1C that during the first three cycles, the WNPI-KC composite gels treated with the ultrasound-TGase combination had lower dehydration rates than the control group. When the ultrasonic power was 200 W, the dehydration rate was the lowest, which indicated a good WHC, and it was consistent with the results of the WHC.

Low-field NMR is used to evaluate the gel quality according to the distribution and binding state of different water molecules [20]. Figure 1D shows the T_2_ decay curves of the composite gels. It can be seen that the WNPI-KC gels have three peaks in the T_2_ curve, which are T_2b_, T_2b-1_ and T_21_, respectively. The T_2b_ and T_2b-1_ peaks (0–100 ms) represent bound water, where T_2b_ represents strongly bound water and T_2b-1_ represents weakly bound water, which can directly interact with the protein group. The T_21_ peak (100–1000 ms) represents immobilized water, which is critical to gel performance. Shorter relaxation time meant that the water molecules were more tightly bound and were less likely to be released. It can be seen from Table 1, compared with the control group, that the T_2b_ peak of U-200W-TG shifted to shorter relaxation time than those of several other samples, indicating that the bound water was more tightly bound to the protein. In addition, according to the proportion of peak area (P_2b_), U-200W-TG had the highest proportion of bound water, indicating that the binding ability of protein and water molecules was stronger under these conditions. Correspondingly, it can be seen from P_21_ that the content of immobilized water in the system decreased, and higher relaxation time T_21_ meant that the water mobility increased, indicating that the immobilized water in the composite system migrated to the bound water.

### 2.2. Effects of Ultrasonic Power on Texture Properties of WNPI-KC Composite Gels

**Figure 2:**
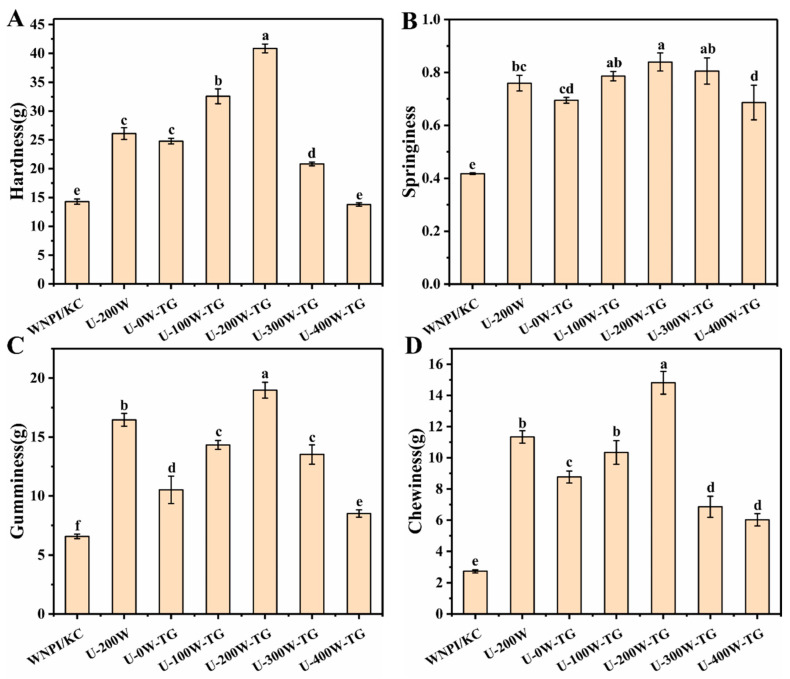
Effects of ultrasonic power on texture properties of WNPI-KC composite gels. (**A**) Hardness; (**B**) Springiness; (**C**) Gumminess; (**D**) Chewiness. Different letters on the bars indicate significant differences (*p* < 0.05).

The texture properties of WNPI-KC gels formed via TGase cross-linking following different ultrasonic power pretreatments are shown in Figure 2. With the increase in ultrasonic power, the hardness, springiness, gumminess and chewiness first increased and then decreased, and they were higher than those of the blank group. This might have been due to the cavitation effect generated by ultrasound, which fully unfolded the structure of WNPI, making it more easy for it to be cross-linked by TGase. Zhang et al. reported that the gel strength of SPI-pectin emulsion gels treated with different ultrasonic power values first increased and then decreased with the increase in ultrasonic power [21]. Zhang et al. investigated that after ultrasonic pretreatment for 40 min, the gel strength of TGase-cross-linked SPI increased from 34.5 to 207.1 g. In this study, the composite gel had the best texture properties when the ultrasonic power was 200 W [18].

### 2.3. Effects of Ultrasonic Power on Rheological Behavior of WNPI-KC Composite Gels

The changes in storage modulus (G’) and loss modulus (G″) of the WNPI-KC composite gel were measured using a rheometer. The value of G’ reflects the solid-like or elastic behavior of the tested material and affects the three-dimensional network structure of gel, while G″ is related to the fluid-like or viscous properties of the material and has no effects on the gel network. G’ was higher than G″, indicating that the gels tended to be more solid. 

**Figure 3:**
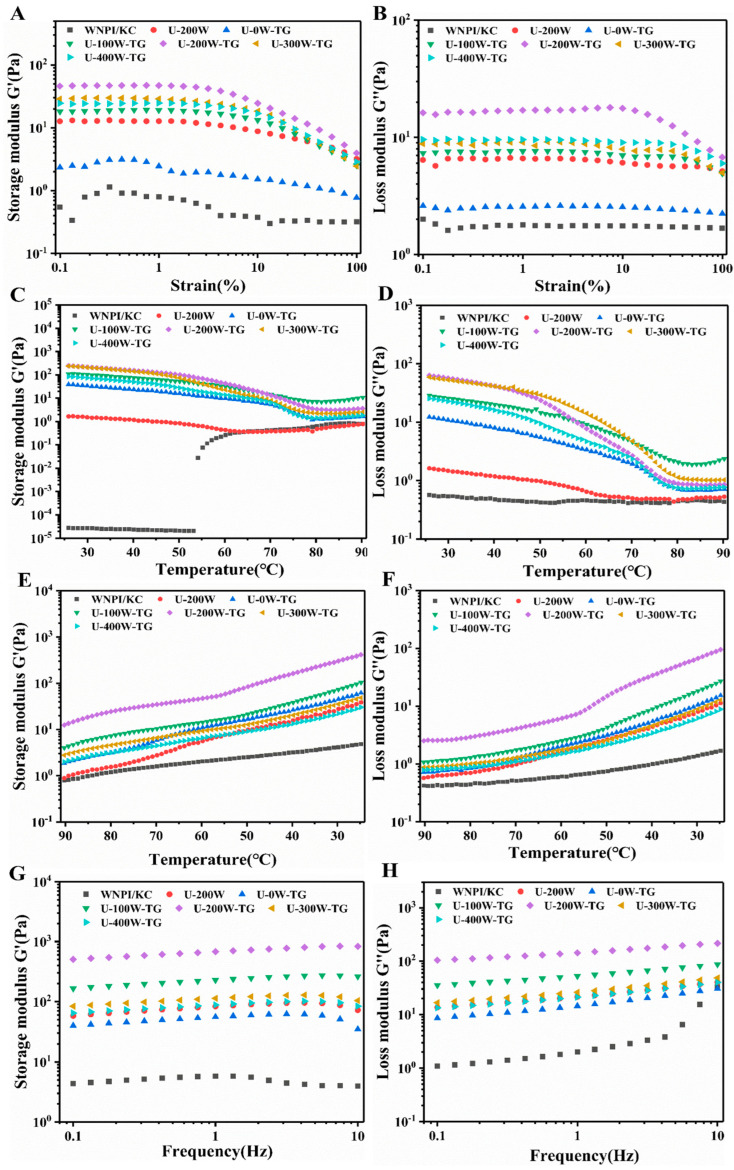
Effects of ultrasonic power on rheological properties of WNPI-KC composite gels. (**A**,**B**) Storage modulus (G’) and loss modulus (G”) of composite gel samples with ultrasound during strain sweep; (**C**,**D**) G’ and G” of the composite gel samples with ultrasound during heating process; (**E**,**F**) G’ and G” of the composite gel samples with ultrasound during cooling process; (**G**,**H**) G’ and G” of the composite gel samples with ultrasound during frequency sweep.

Figure 3A,B show G’ and G″ of the gels under stress–strain sweeps. All gel samples showed similar behavior, and the change values of G’ and G″ were independent of strain at smaller strains and exhibited linear viscoelastic behavior, which was also the reason for the 1% strain chosen in the temperature and frequency sweep experiments. G’ and G″ decreased with the increase in strain, indicating typical solid-like gel, which is also found in soy protein isolate gels [22]. As the strain further increased, G’ and G″ intersected, and the stress at this time was considered to be fracture stress, indicating that the gel structure began to break down. With the increase in ultrasonic power, G’ of the gel increased and was significantly higher than G″, and a larger amount of deformation was required to break the gel network, indicating that ultrasonic treatment could improve the gel properties. The gel stability was the best with 200 W ultrasonic power.

The viscoelastic properties of WNPI-KC composite gels treated using different ultrasonic power values were characterized with dynamic rheological measurements during heating–cooling cycles (Figure 3C–F). G’ was consistently higher than G″ during heating and cooling cycles, indicating that the gels exhibited better elastic characteristics. During the cooling scan, G’ of all gels increased continuously, which might have been related to the gradual stabilization of the gels. Sun and Arntfield found that during the cooling scan, the formed protein gel network structure was mainly maintained by hydrogen bonds and hydrophobic interactions [23]. When sonicated at 200 W, the WNPI-KC composite gel had the highest G’ value.

The frequency sweep of WNPI-KC composite gels was performed after the temperature sweep. As shown in Figure 3G,H, the moduli of individual proteins and polysaccharides were small, and no gel was basically formed. Following treatment with ultrasound and TGase, G’ and G″ increased, showing an enhanced gelation effect. G’ was always greater than G″ in all samples, showing good solid-like properties. When the ultrasonic power was 200 W, G’ of the composite gel was the highest, indicating that a good three-dimensional network structure had formed, which was consistent with the observation of SEM. Studies showed that most heat-induced plant protein gels are weak gels, mainly formed by non-covalent bonds, such as electrostatic interactions, hydrogen bonds, hydrophobic interactions, etc. [24].

### 2.4. Effects of Ultrasonic Power on D [4,3], Zeta Potential and Apparent Viscosity of WNPI-KC Composite Gels

**Figure 4:**
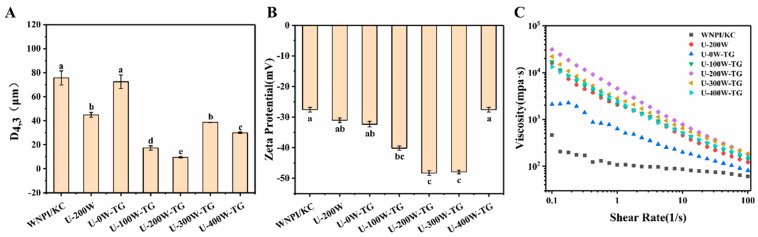
Effects of ultrasonic power on D [4,3] (**A**), zeta potentials (**B**) and apparent viscosity (**C**) of WNPI-KC composite gels. Different letters indicate significant differences (*p* < 0.05).

The D [4,3] and zeta potentials of TGase-cross-linked gels subjected to different ultrasonic power values are shown in Figure 4A. The particle sizes of all samples were between 10 and 100 μm, and the D [4,3] of WNPI-KC was significantly reduced with ultrasonic pretreatment. Zeta potential can be used to study changes in protein conformation through electrostatic interactions [25]. As shown in Figure 4B, the zeta potentials of all samples were negative. After ultrasonic pretreatment, the absolute value of the zeta potential of each sample increased significantly, and that of U-200W-TG was the highest. This might have been because more negatively charged groups were exposed on the WNPI surface after sonication. A higher electrostatic force can help the protein to maintain a stable gel network, so when the ultrasonic power was 200 W, the composite system was the most stable.

The changes in apparent viscosity with the shear rate are shown in Figure 4C. The apparent viscosity of all samples decreased with the increase in the shear rate and exhibited typical pseudoplastic and non-Newtonian mechanical behavior in the shear rate range of 0.1–100 s^−1^ [26]. The apparent viscosity of WNPI-KC induced by sonication alone and TGase-cross-linked alone was higher than that of the blank group, and following the two combined treatments, it was the highest. Ultrasonic pretreatment significantly increased the apparent viscosity of TGase-cross-linked WNPI-KC, which might have been due to the higher apparent viscosity caused by the high-molecular-weight polymer formed after cross-linking [27]. However, as the ultrasonic power increased from 200 to 400 W, the apparent viscosity of TGase-cross-linked WNPI-KC decreased slightly. This might have been due to the fact that with the increase in ultrasonic power leading to the formation of larger polymers, high-molecular-weight polymers did not change significantly after the cross-linking reaction.

### 2.5. Effects of Ultrasonic Power on the Structural Properties of WNPI-KC Composite Gels

#### 2.5.1. Microstructure

**Figure 5:**
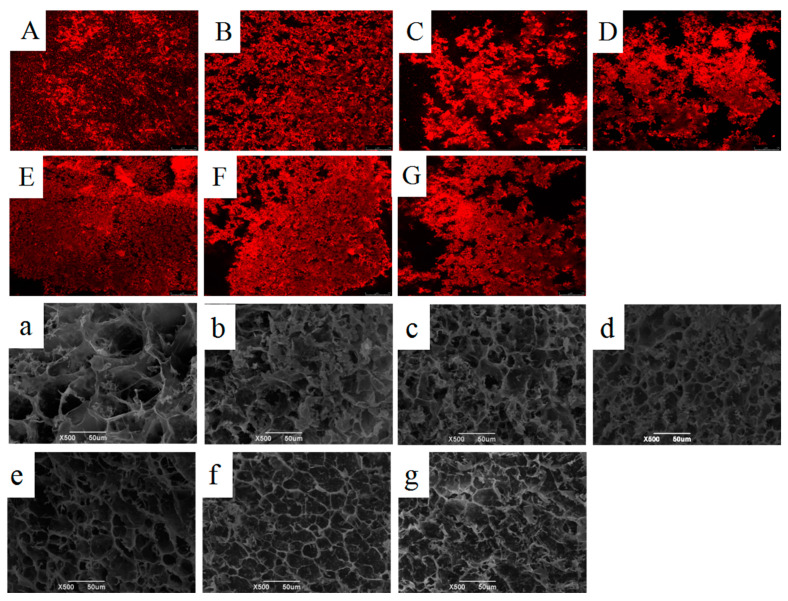
Effects of ultrasonic power on CLSM images (**A**–**G**: WNPI/KC, U-200W, U-0W-TG, U-100W-TG, U-200W-TG, U-300W-TG and U-400W-TG) and SEM images (**a**–**g**: WNPI/KC, U-200W, U-0W-TG, U-100W-TG, U-200W-TG, U-300W-TG and U-400W-TG) of WNPI-KC composite gels.

The microstructure of a gel system can help to define the physical properties of the gel structure. Figure 5A,G show the laser scanning confocal microscope images of the composite gels. When subjected to the low ultrasonic power values of 0 and 100 W, the composite gel experienced an obvious aggregation phenomenon, while after 200 and 300 W ultrasonic treatment, the aggregation of the composite gel was obviously improved, and it was composed of uniform small particles. This might have been because more hydrophobic groups were exposed on WNPI after sonication, which is conducive to the gelation of WNPI and KC [28]. In addition, proper ultrasonic power improved the homogeneity of the composite gel, allowing proteins to have a larger surface area to aggregate with polysaccharides, thereby forming a denser gel network structure.

The network structure of gel can be visualized using SEM. It can be seen in Figure 5a that the WNPI-KC composite gel had larger pores, while the gel pores were significantly reduced after ultrasonic treatment and TGase treatment alone (Figure 5b,c), but the surface of the gels was still rough. After ultrasonic-TGase combined treatment, the gel network structure was more compact, and when the ultrasonic power was 200 W, the gel network structure was the best, which was beneficial to the improvement of gel strength and WHC (Figure 5e). This might have been because ultrasound promoted the unfolding of protein structures and the cross-linking of TGase to form a uniform and dense gel network [29]. In addition, such phenomenon was also consistent with the texture, rheological properties and WHC of WNPI-KC gel. However, excessive sonication led to the reaggregation of WNPI, reducing the solubility of WNPI. This resulted in a rough gel network structure, and WHC and gel strength were affected accordingly.

#### 2.5.2. FTIR

**Figure 6:**
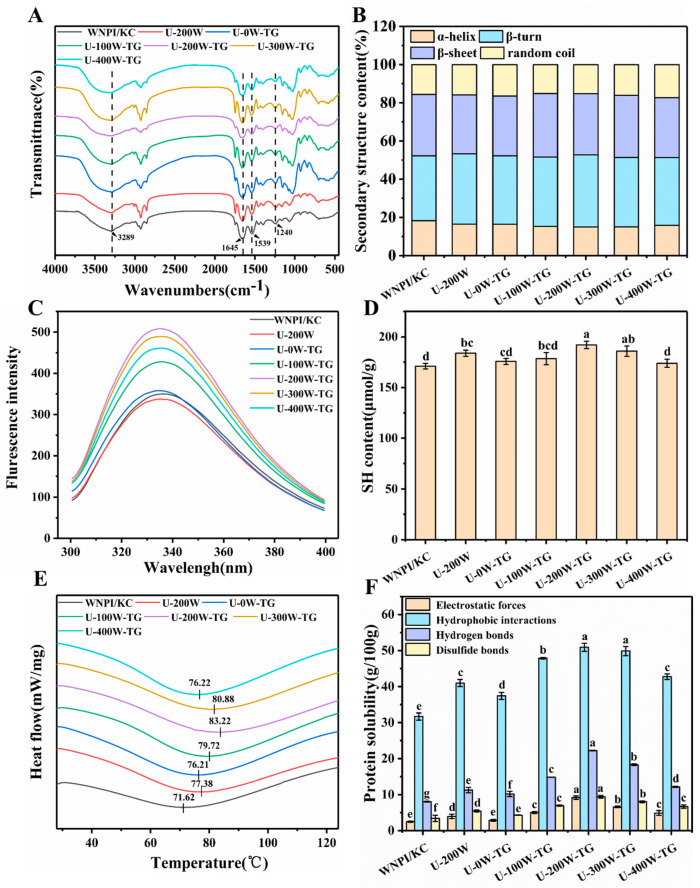
Effects of ultrasonic power on infrared spectra (**A**), secondary structure contents (**B**), fluorescence intensities (**C**), SH contents (**D**), thermal stability (**E**) and intermolecular forces (**F**) of WNPI-KC composite gels. Different letters on different color bars indicate significant differences (*p* < 0.05).

FTIR can be used to monitor the interactions between proteins and polysaccharides. The infrared spectra of composite gels are shown in Figure 6A. In general, the characteristic absorption peaks of the composite gel after ultrasonic-TGase treatment are the same as those of the untreated gel, indicating that no new functional groups were generated. A strong and broad absorption peak (3289 cm^−1^) is observed at 3200–3600 cm^−1^ in all composite gels, which is consistent with research on soy protein isolate–Smilax china L. starch composite gel [30]. The bands in this region represent the N-H stretching vibration of intermolecular hydrogen bonds of proteins and the O-H stretching vibration of polysaccharides and proteins, indicating the formation of hydrogen bonds [31]. There is also a weak peak at about 2927 cm^−1^, which is related to C-H stretching vibration [32]. 

The amide I band (1600–1700 cm^−1^) is considered a reliable indicator of protein secondary structure conformation [33]. As shown in Figure 6B, the α-helix content of the composite gel decreased, and the *β*-sheet content increased with the increase in ultrasonic power. The transition of α-helices to *β*-sheets promotes gelation [34]. Chen et al. found that the addition of guar gum to SPI increased *β*-sheet content, resulting in better gel strength and WHC [35].

#### 2.5.3. Intrinsic Fluorescence Spectroscopy

The intrinsic fluorescence spectrum can reflect the tertiary structure of a protein through the change in the polarity of the hydrophobic amino acid microenvironment of the protein [36]. As shown in Figure 6C, the fluorescence intensity of the control group was the lowest. After ultrasonic and TGase treatment, the fluorescence intensity increased, and after the two combined treatments, it was further improved. This showed that the complex treatment could promote the unfolding of the protein structure, reduce the interaction of the tryptophan group and the solvent or protein and thus increase the fluorescence intensity [37]. In addition, the change in the polarity of the microenvironment usually leads to a shift in the wavelength of the highest emission peak, but the change observed in this study was not very obvious, and the same phenomenon was also reported by Li et al. [38].

#### 2.5.4. Free Sulfhydryl Content

Changes in free sulfhydryl content can reflect the degree of protein denaturation. As shown in Figure 6D, compared with the control group, both ultrasonic treatment and TGase treatment could significantly increase the free sulfhydryl content, and this was further enhanced after ultrasonic-TGase combined treatment, showing a synergistic effect. The reason for this phenomenon might be that the ultrasonic treatment unfolded the protein structure and exposed more free sulfhydryl groups [39]. In addition, the combined sonication-TGase treatment might have led to the breaking of the intramolecular disulfide bonds of the protein, thereby converting them into free sulfhydryl groups. However, when the ultrasonic power was 400 W, the content of free sulfhydryl groups decreased, which might have been due to excessive sonication having reaggregated protein molecules to form larger protein aggregates, leading to the decrease in the content of free sulfhydryl groups.

#### 2.5.5. Thermal Stability

Figure 6E shows the thermal denaturation temperature of WNPI-KC composite gels following different ultrasonic power pretreatments. Ultrasonic pretreatment increased the thermal transition temperature of WNPI-KC gels, and it reached a peak value of 83.22 °C when the ultrasonic power was 200 W. However, too-high ultrasonic power can lead to the destruction of the gel network. Therefore, when the ultrasonic power reached 300 and 400 W, the thermal transition temperature decreased, which was related to the gel strength and gel viscoelasticity. The results showed that the thermal stability of the composite gel could be enhanced using appropriate ultrasonic power, which might have been due to the enhanced intermolecular interaction forces in the gel induced by ultrasound.

#### 2.5.6. Intermolecular Forces 

Assessing gel solubility in different solvents reveals the types of protein interactions. The hydrophobic interactions were highest in the composite gels, suggesting that the hydrophobic interactions contributed the most to gel formation (Figure 6F). Ultrasonic-TGase treatment could cause protein denaturation and the exposure of hydrophobic groups, promoting protein cross-linking and aggregation, thereby enhancing the integrity of the gel network. Related studies showed that hydrophobic interactions could promote the formation of regular protein aggregates. Wang et al. showed that the interaction in Mesona blumes polysaccharide-SPI mixed gel was hydrophobic interaction [6]. Combined treatment with ultrasound-TGase was favorable for the formation of hydrogen bonds. Cao et al. found that with the increase in hydrogen bond interaction, the gel strength of surimi gel increased [40]. Disulfide bonds were also significant in heat-induced protein gel formation. Combined ultrasonic-TGase treatment significantly increased the disulfide bond content of the composite gel, which improved composite gel properties. Jiang et al. documented that the addition of Mesona chinensis polysaccharides promoted the formation of disulfide bonds in WPI gels and formed a stable gel network [41].

### 2.6. Schematic Mechanism

**Figure 7:**
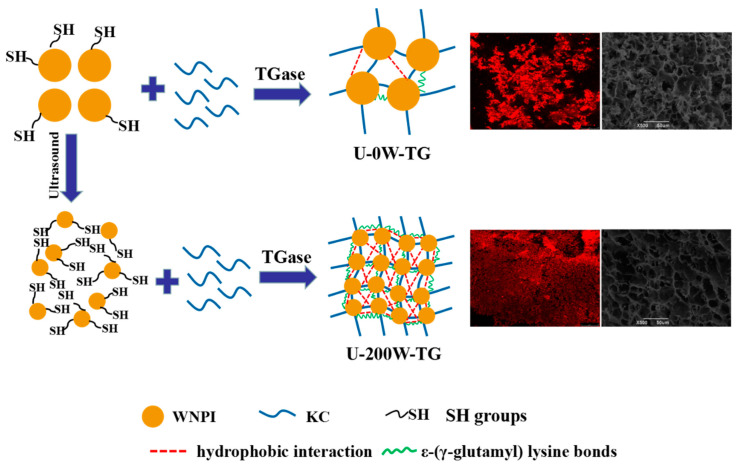
Schematic mechanism of WNPI-KC gel formation after treatment with ultrasound.

The schematic mechanism of the effect of ultrasonic pretreatment on the TGase-cross-linked WNPI-KC composite gel is shown in Figure 7. The structure properties of WNPI were altered due to the cavitation phenomenon induced by ultrasound. Sonicated WNPI could be better dispersed in aqueous solution, and hydrophobic residues and free SH could be exposed on the surface of WNPI molecules. Sonicated WNPI was a good substrate for TGase cross-linking, which could better cross-link with KC. At the same time, non-covalent interactions and intermolecular ε-(γ-glutamine) lysine groups were enhanced, which contributed to form a good, complex gel network. The above changes led to a more uniform and much denser microstructure of WNPI-KC composite gel after ultrasonic treatment, and its gel strength, WHC and rheological properties were improved. By contrast, many functional groups, such as free sulfhydryl groups and hydrophobic residues, were still buried inside the protein molecule and thus were not easily cross-linked by TGase in unsonicated WNPI, resulting in fewer intermolecular ε-(γ-glutamine) lysine groups and non-covalent interactions; so, the gel performance was poor. 

## 3. Conclusions

In this study, the effects of ultrasonic pretreatment on the structure, and physicochemical and gel properties of TGase-cross-linked WNPI-KC composite gels were investigated. The results showed that 200 W ultrasonic power could significantly promote the transformation of α-helices into β-folds in protein molecules and increase the fluorescence intensity and the content of free sulfhydryl groups, which led to the formation of a uniform, dense gel network. Further, 200 W ultrasonic power could also enhance the intermolecular forces between WNPI and KC, making them more closely combined with water molecules. Under these conditions, the texture properties, rheological properties, WHC and thermal stability of WNPI-KC gel were the best, while excessive ultrasonic power (300–400 W) made WNPI protein molecules aggregate, which was not conducive to the improvement of gel properties. This experiment shows the construction of a new ultrasound-TGase-regulated WNPI gel system, providing good technical support for the application of WNPI gel food. In the future, the mechanism of action and practical application scenarios need to be further studied. 

## 4. Materials and Methods

### 4.1. Materials 

Walnuts were supplied by a market (Wuhan, China). TGase was obtained from Shandong Gushuo Biotechnology Co., Ltd. (Jining, China). κ-Carrageenan was bought from Henan Chinuo Food Ingredients Co., Ltd. (Zhengzhou, China). β-mercaptoethanol was purchased from Macklin Biochemical Technology Co. (Shanghai, China). The other chemicals used were of analytical grade.

### 4.2. Preparation of WNPI/KC Composite Gels

WNPI was extracted on the basis of our previous method [42]. Specifically, 10% (*w/v*) stock solution of WNPI was stirred at room temperature for 2 h and then pretreated using different ultrasonic power values (0 W, 100 W, 200 W, 300 W and 400 W; sonication time of 20 min). According to the same method, 1% (*w/v*) KC stock solution was obtained, and the ultrasonically pretreated WNPI stock solution and KC stock solution were uniformly mixed in a ratio of 1:1 (*v/v*). We added 2% (*w/v*) TGase to each mixture, allowed the mixtures to react at 45 °C and pH 4.5 for 2 h and then heated them in a water bath at 90 °C for 30 min. After immediate cooling, we stored them at 4 °C for 12 h.

### 4.3. Water-Holding Capacity (WHC)

A 5 g gel sample was weighed and centrifuged at 8500 r/min for 15 min. The WHC is defined as the percentage of gel mass after centrifugation to initial gel mass [43].

(1) $$\mathrm{WHC}(\%)=\frac{W_{2}-W_{0}}{W_{1}-W_{0}}\times 100$$

where *W_0_* is the mass of the empty centrifugal tube (g), *W_1_* is the total weight of sample and centrifugal tube before centrifugation (g) and *W_2_* is the weight of sample and tube after centrifugation with supernatant removed (g).

### 4.4. Swelling Capacity

The swelling capacity of the composite gel was determined using the method of Feng et al. with minor modifications [44]. A certain quantity of gel was cut and immersed in phosphate buffer (0.1 M, pH 7.0). The swollen gel sample was taken out of the buffer after soaking for 24 h; then, excess water was filtered out and weighed. The calculation formula of the swelling capacity is the following:

(2) $$\mathrm{Swelling}\;\mathrm{ratio}\;(\%)=\frac{W_{2}-W_{1}}{W_{1}}\times 100$$

where *W_1_* is the mass of the initial gel and *W_2_* is the mass of the swollen gel.

### 4.5. Freeze–Thaw Stability

The freeze–thaw stability was measured with the method described by Liang et al. [45]. The WNPI-KC composite gel was frozen at −20 °C for 22 h, thawed at 30 °C for 2 h, and centrifuged at 8000 r/min for 10 min to remove the released water. The above operation was repeated 5 times. The syneresis rate was calculated as follows:

(3) $$\mathrm{Syneresis}\;\mathrm{rate}\;(\%)=\frac{W_{n}-W_{n\prime }}{W_{n}-W_{0}}\times 100$$

where *W_0_*, *W_n_* and *W_n′_* are the mass of the empty centrifuge tube, the total mass of centrifuge tube and gel, and the total mass after centrifugation, respectively; and *n* is the number of cycles.

### 4.6. Moisture Distribution

Gel samples treated using different ultrasonic power values were placed in glass tubes (25 mm in diameter), and T_2_ relaxation times were measured with a MesoMR23-060H-I LF-NMR analyzer. Peak areas were calculated with corresponding signal scoring and data analysis [46].

### 4.7. Texture Properties

The texture of WNPI-KC gel (15 mm in height and 30 mm in diameter) was analyzed with a texture analyzer. Hardness, elasticity and adhesion were measured with a P/0.5R cylindrical probe. Using the secondary compression method, the compression degree was 30%. The trigger force was set at 5 g, and the pre-test, test and post-test speeds were set at 1.0 mm/s, 0.5 mm/s and 1.0 mm/s, respectively.

### 4.8. Rheological Properties

The dynamic rheological properties of WNPI-KC gel were measured using an Anton Paar rheometer according to Alavi et al. with some modifications [47]. First, a stress–strain sweep was performed; the temperature was set at 25 °C; the fixed frequency was 1 Hz; and the shear strain was from 0.1 to 100%. The sample was heated from 25 to 90 °C and then cooled from 90 to 25 °C at the same rate. After the temperature sweep was completed, a frequency sweep was performed by applying an angular frequency of 0.1–10 rad/s at a fixed strain of 1%. The shear rate was 0.01 s^−1^–100 s^−1^ when measuring the apparent viscosity, and the logarithmic mode was set to collect several points.

### 4.9. Zeta Potential and Particle Size

The zeta potential of the samples was determined with a nanoparticle-size potentiometer; we diluted the sample by 50 times and performed measurements after equilibration for 120 s. The D [4,3] of the samples was tested using a Malvern particle-size analyzer with pump speed set at 2500 r/min.

### 4.10. Microstructure

The microstructure of the samples was observed using CLSM. The composite gel was stained with 0.02% rhodamine B, and the excitation wavelength was set at 546 nm.

The surface morphology of the samples was observed using an SU-8010 SEM. The freeze-dried WNPI-KC composite gel was glued to the sample stage, and 10 nm gold films were coated using ion sputtering and then observed using scanning electron microscopy at 15.0 kV and magnifications of 100× and 500×.

### 4.11. Fourier Transform Infrared Spectroscopy (FTIR)

The samples were pressed with potassium bromide in a mass ratio of 1:250 and scanned using FTIR. In total, 64 scans were made in the scanning range of 400–4000 cm^−1^.

### 4.12. Intrinsic Fluorescence Spectroscopy

Fluorescence spectrum scanning was performed on WNPI solutions (1 mg/mL) treated with different ultrasonic power values using a fluorescence spectrophotometer. The excitation wavelength, scan range and scan speed were set at 280 nm, 300–400 nm and 1200 nm/min, respectively.

### 4.13. Free Sulfhydryl Content

A sample solution at a concentration of 1 mg/mL was prepared with triglycine, and 40 μL of DTNB solution (4 mg/mL) was added to the 4 mL sample, mixed well and allowed to react in the dark for 30 min [48]. Absorbance was measured at 412 nm, and the reagent blank was the control group. The free sulfhydryl content was calculated with the following formula:

(4) $$\;\mathrm{Sulfhydryl}\;(\mu \mathrm{mol}/100\;\mathrm{mg})=\frac{A_{412}{\times D\times 10}^{5}}{{13,600\times C}_{\mathrm{pr}}}$$

where *A*_412_ is the absorbance value at 412 nm, *D* is the dilution factor, *C_pr_* is the sample concentration (mg/mL) and 13,600 is the absorbance coefficient.

### 4.14. Thermal Stability

The thermal transition temperature of the composite gel was determined using Mettler-Toledo DSC1 differential scanning calorimetry. A 3–5 mg sample of the lyophilized gel was weighed and placed in an aluminum crucible. The measurement was carried out in the temperature range of 25 to 125 °C.

### 4.15. Intermolecular Forces

The samples treated with different ultrasonic power values were dispersed in different solvents based on the method of Lei et al. [49]. In total, 0.5 g of gel sample was thoroughly mixed with buffer, allowed to react for 20 min, and centrifuged to take the supernatant, and the soluble protein content in the supernatant was determined using the biuret method; all samples were measured three times [50].

### 4.16. Statistical Analysis

Origin 8.0 software was used for graphing. The significance level for differences between means was set at *p* < 0.05 (Tukey’s test) using SPSS software.

## Figures and Tables

**Table 1:** Effects of ultrasonic power on transverse relaxation time and peak area of WNPI-KC composite gels. Different letters in the same column indicate significant differences (*p* < 0.05).

	T_2b_ (ms)	T_2b−1_ (ms)	T_21_ (ms)	P_2b_ (%)	P_2b−1_ (%)	P_21_ (%)
WNPI/KC	2.42 ± 0.65 ^ab^	23.28 ± 0.92 ^a^	420.10 ± 17.03 ^a^	0.45 ± 0.23 ^ab^	1.66 ± 0.19 ^a^	97.89 ± 0.12 ^ab^
U-200 W	0.55 ± 0.32 ^c^	8.82 ± 0.72 ^bc^	333.13 ± 0.00 ^bc^	0.25 ± 0.20 ^b^	1.06 ± 0.08 ^bc^	98.69 ± 0.13 ^a^
U-0 W-TG	2.84 ± 1.86 ^a^	21.26 ± 1.66 ^a^	357.08 ± 0.00 ^b^	0.39 ± 0.13 ^ab^	1.23 ± 0.13 ^b^	98.38 ± 0.04 ^ab^
U-100 W-TG	0.47 ± 0.08 ^c^	10.60 ± 0.43 ^bc^	357.08 ± 0.00 ^b^	0.65 ± 0.50 ^ab^	1.10 ± 0.11 ^bc^	98.25 ± 0.47 ^ab^
U-200 W-TG	0.31 ± 0.13 ^c^	10.86 ± 3.92 ^b^	357.65 ± 24.81 ^b^	1.63 ± 1.29 ^a^	0.93 ± 0.13 ^c^	97.44 ± 1.32 ^b^
U-300 W-TG	0.94 ± 0.28 ^bc^	9.51 ± 1.41 b^c^	318.23 ± 12.90 ^c^	0.67 ± 0.20 ^ab^	1.02 ± 0.19 ^bc^	98.31 ± 0.04 ^ab^
U-400 W-TG	--	7.15 ± 0.28 ^c^	349.10 ± 13.83 ^b^	--	1.10 ± 0.08 ^bc^	98.90 ± 0.08 ^a^

## Data Availability

Not applicable.
